# Distinct Functions of Endophilin Isoforms in Synaptic Vesicle Endocytosis

**DOI:** 10.1155/2015/371496

**Published:** 2015-11-22

**Authors:** Jifeng Zhang, Minghui Tan, Yichen Yin, Bingyu Ren, Nannan Jiang, Guoqing Guo, Yuan Chen

**Affiliations:** ^1^Department of Anatomy, Medical College of Jinan University, Guangzhou 510630, China; ^2^Center for Neurobiology, Zhongshan School of Medicine, Sun Yat-Sen University, Guangzhou 510080, China; ^3^Department of Orthopedics, The First Affiliated Hospital of Jinan University, Guangzhou 510630, China; ^4^Department of Neurology, Guangzhou Red Cross Hospital, The Fourth Affiliated Hospital of Jinan University, Guangzhou 510220, China

## Abstract

Endophilin isoforms perform distinct characteristics in their interactions with N-type Ca^2+^ channels and dynamin. However, precise functional differences for the endophilin isoforms on synaptic vesicle (SV) endocytosis remain unknown. By coupling RNA interference and electrophysiological recording techniques in cultured rat hippocampal neurons, we investigated the functional differences of three isoforms of endophilin in SV endocytosis. The results showed that the amplitude of normalized evoked excitatory postsynaptic currents in endophilin1 knockdown neurons decreased significantly for both single train and multiple train stimulations. Similar results were found using endophilin2 knockdown neurons, whereas endophilin3 siRNA exhibited no change compared with control neurons. Endophilin1 and endophilin2 affected SV endocytosis, but the effect of endophilin1 and endophilin2 double knockdown was not different from that of either knockdown alone. This result suggested that endophilin1 and endophilin2 functioned together but not independently during SV endocytosis. Taken together, our results indicate that SV endocytosis is sustained by endophilin1 and endophilin2 isoforms, but not by endophilin3, in primary cultured hippocampal neurons.

## 1. Introduction

Clathrin-mediated endocytosis (CME) is an evolutionarily conserved process that cells use to internalize specific components of the plasma membrane [1, 2]. In neurons, CME plays a particularly important role, functioning both on the presynaptic and postsynaptic sides of neuronal synapses [3, 4]. Numerous studies have revealed that CME regulates the recycling of synaptic vesicles (SVs) at presynaptic membranes [4, 5], mediates the internalization of neurotransmitter receptors, and contributes to synaptic plasticity by controlling postsynaptic excitability at postsynaptic membranes [5–8].

Endophilin is one of the major endocytic proteins important in CME [9–11]. At least three isoforms of endophilin have been identified and distributed in various tissues. For instance, endophilin1 is expressed only in the brain, endophilin2 exists in multiple tissues, and endophilin3 is found mainly in the brain and testis [12–14]. Endophilin dysfunction has been linked to both cancer and neurodegenerative diseases [9, 15–17]. Microinjection of endophilin antibodies into giant axons causes a stimulus-dependent depletion of SV in lamprey [10]. Mutant* Drosophila* larvae lacking endophilin fail to take up FM1-43 dye, indicating an inability to retrieve synaptic membranes [18]. Endophilin1 assists in downregulating epidermal growth factor receptor (EGFR) and other growth factor receptors [15]. Similarly, overexpression of endophilin2 in 293T cells increases EGF-induced endocytosis of EGFRs [19]. However, endophilin3 reportedly inhibits receptor-mediated endocytosis. The COS-7 cells transfected with endophilin1 do not affect transferrin uptake efficiency, whereas transfection with full-length endophilin3 strongly reduces it [20].

At the subcellular level, all three endophilin isoforms are concentrated at synaptic terminals and in the cytosol of neurons [13]. Electron microscopy of immunogold-labeled endophilin shows that endophilin is localized at both presynaptic and postsynaptic membranes. Endophilin functions as a membrane-bending molecule and is delivered to endocytic zones by exocytosis [21]. Endophilin2 and endophilin3, but not endophilin1, are involved in Arc/Arg3.1-mediated AMPA receptor endocytosis on postsynaptic membranes, suggesting that isoform specificity can confer particular properties to specific endocytic pathways [21]. At the presynaptic terminal, a series of studies revealed that the functional domains of endophilin affect several stages of CME [9, 10]. An amino acid sequence analysis showed that all three endophilin isoforms consist of an N-terminal Bin/amphiphysin/Rvs (BAR) domain, a variable middle region, and a C-terminal SRC homology-3 (SH3) domain [22]. The BAR domain mainly participates at the base of the membrane invagination, an early step in endocytosis of the membrane [23]. The SH3 domain is involved in the interaction of endophilin with other endocytic machinery molecules that contain a proline-rich domain (PRD), such as synaptojanin and GTPase dynamin [12–14]. Disrupting the interactions perturbs the fission and uncoating of synaptic clathrin-coated vesicles [24]. Our previous studies found an unconventional PRD embedded within endophilin2, and the intramolecular interaction between this unconventional PRD and the C-terminal SH3 domain is Ca^2+^-dependent [25]. Further biochemical studies demonstrated that endophilin1 has a similar Ca^2+^-dependent interaction with voltage-gated Ca^2+^ channels and dynamin as endophilin2, whereas endophilin3 does not [26]. These findings imply that these structurally similar endophilin isoforms may have distinct characteristics in interactions with other endocytic machineries. However, the precise functional differences of each endophilin isoform on presynaptic SV endocytosis remain unknown.

Here, we report that endophilin isoforms have different effects on SV endocytosis. We found that SV endocytosis was impaired in endophilin1 or endophilin2 knockdown neurons but was unaffected in endophilin3 knockdown neurons. Our results indicate that endophilin1 or endophilin2, but not endophilin3, is the main regulatory machinery involved in clathrin-mediated SV endocytosis.

## 2. Materials and Methods

### 2.1. Ethics Statement

The experiments were conducted with one-day-old Sprague Dawley rats. All animal procedures were performed in strict accordance with the recommendations in the* Guide for the Care and Use of Laboratory Animals* produced by the National Institutes of Health. The protocol was approved by the Institutional Animal Care and Use Committee at Zhongshan School of Medicine in Sun Yat-Sen University. All efforts were made to minimize the suffering and number of animals used.

### 2.2. Hippocampal Neuronal Culture and Transfection

Rat hippocampal neurons were cultured as described previously [27]. Neurons cultured in 24-well culture plates at 12–14 days* in vitro* were used to perform the transfection. Endophilin siRNA (100 pmol) was combined with 37 *μ*L of 2.5 M CaCl_2_ solution in sterile, deionized water for a final volume of 300 *μ*L and then mixed well with 300 *μ*L of 2x HEPES-buffered saline. The mixture was vortexed and incubated at 25°C for 15 min. In each well, 30 *μ*L of the mixture was added dropwise to the cells and allowed to incubate for 40 min. A GFP expression plasmid was cotransfected with the siRNAs to mark the transfected cells.

### 2.3. Plasmids

The full-length endophilin1, endophilin2, and endophilin3 cDNA fragments were subcloned into pEGFP-C1 plasmids (Clontech). All constructs were verified by sequencing. A detailed description of the methods used for constructing cDNA plasmids is available in our previous studies [25, 26].

### 2.4. RNA Interference

The 19-nucleotide siRNAs for each endophilin isoform and their related negative controls (NC, scrambled sequence) were designed using online software (http://www.promega.com/siRNADesigner/) and were synthesized by Shanghai Gene-Pharma Co., Ltd. (Shanghai, China). These included the si1-1- and si1-2-targeted sequences 5′-GGGCTAAACTCAGTATGAT-3′ and 5′-CCGACGCTTAGACTTTGAT-3′ of endophilin1 mRNA (NM_053935), si2-1- and si2-2-targeted sequences 5′-GAGGTTCTATTACCTTTCT-3′ and 5′-GCTTCGTCATCATTTAGAT-3′ of endophilin2 mRNA (NM_031239), and si3-1- and si3-2-targeted sequences 5′-GCCGAAAGAAGCATGTTTA-3′ and 5′-GCTTCGTCATCATTTAGAT-3′ of endophilin3 mRNA (NM_031238). The targeted regions showed no significant homology with any other genes using BLAST.

### 2.5. HEK293 Cell Culture and Transfection

Human embryonic kidney (HEK) 293 cell culture was performed as described previously [27]. To determine the efficacy and specificity of siRNAs, cotransfection of 100 pmol of siRNAs or NC together with 2 *μ*g of the corresponding endophilin-pEGFPC1 plasmids into HEK293 cells was performed using calcium phosphate.

### 2.6. Western Blotting

The cells were lysed and separated 48 h after cotransfection using 10% SDS-PAGE and electrophoretically transferred to PVDF membranes (Pierce, Rockford, IL). The membranes were blocked with 5% nonfat milk in TBS and 0.1% Tween-20 and then incubated with an isoform-specific endophilin antibody or anti-endophilins1–3 antibody (Santa Cruz). After three to four washes with TBS and 0.1% Tween-20, the membranes were incubated with HRP-conjugated secondary antibodies (Jackson ImmunoResearch). The protein bands were detected after developing the blots using an ECL kit (Pierce).

### 2.7. Immunostaining

Three days after siRNA transfection, the hippocampal neurons were fixed with 4% paraformaldehyde (Sigma). Immunostaining was then performed using a standard protocol described previously [27]. The primary antibodies, anti-endophilin1, 2, or 3, were used at a dilution of 1 : 100, and donkey anti-goat IgG (H + L) Dylight 549 (Jackson ImmunoResearch) was used at a dilution of 1 : 500. After staining, the cells were mounted on glass slides using Fluoro Gel II with DAPI (EMS) and imaged with a Carl Zeiss LSM 710 confocal microscope. Images were acquired with the same optical slice thickness in every channel using a 63x oil objective and a resolution of 1024 × 1024 pixels. The RNA interference efficiency in hippocampal neurons was determined by calculating the percentage of endophilin-positive cells, as previously described [28].

### 2.8. Electrophysiological Recordings

Excitatory postsynaptic currents (EPSCs) were recorded in cultured hippocampal neurons using dual whole-cell recordings by evoking an action potential (AP) or a train of APs in transfected neurons. The APs were evoked in transfected neurons by delivering brief (3 ms) and large (300–400 pA) depolarizations in current-clamp mode, and EPSCs were recorded in voltage-clamp (−70 mV) mode in a nearby, nontransfected neuron. For the input-output relationship of APs, APs were evoked using a 400 ms current injection at various intensities (0–200 pA). To measure the depletion of the readily releasable pool (RRP) of SVs, the transfected neurons were challenged with high-frequency stimulation (100 pulses at 5 Hz). To compare the size of the frequency-dependent depression of EPSCs during high-frequency stimulation, the 2nd to 100th responses were normalized to the 1st response, and the last 20 responses were averaged and normalized to the 1st response to compare among the groups. To measure the replenishment of the RRP, the transfected neurons were stimulated with consecutive high-frequency stimulation. For consecutive high-frequency stimulation, four trains of stimulation (5 Hz, 100 stimuli) were applied with a 3 min interval, during which the transfected neuron was stimulated at 0.1 Hz. To compare the recoveries of the RRP, the EPSC amplitudes recorded at 0.1 Hz after the 1st, 2nd, 3rd, and 4th train were normalized to the amplitude recorded before the 1st train. The experiment was conducted at room temperature using an EPC-10 patch-clamp amplifier and Patchmaster software (Heka Electroniks). The EPSCs were recorded with a patch electrode (3–5 MΩ tip resistance) and filtered at 2 kHz. The extracellular bath solution contained (in mM) NaCl, 128; KCl, 5; CaCl_2_, 2; MgCl_2_, 1; glucose, 15; and HEPES, 20 (pH 7.3). The pipette solution contained (in mM): KCl, 147; Na_2_-phosphocreatine, 5; EGTA, 2; MgATP, 2; Na_2_GTP, 0.3; and HEPES, 10 (pH 7.2).

### 2.9. Statistical Analysis

Data are presented as the mean ± SEM. The statistical significance of the differences was analyzed using Student's *t*-test between two groups and one-way ANOVA with Newman-Keuls* post hoc* tests for comparisons among more than two groups.

## 3. Results

### 3.1. Knockdown of Endophilin Isoforms Does Not Affect SV Exocytosis

**Figure 1:**
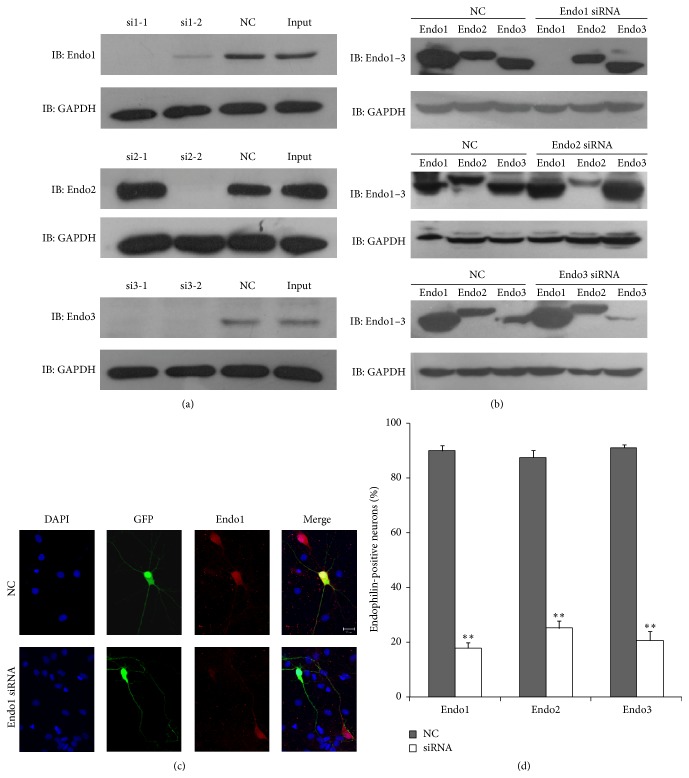
Efficacy and specificity of the designed siRNAs. (a) Immunoblotting detection of endophilin isoforms and GAPDH in HEK293 cells cotransfected with endophilin-pEGFPC1 plasmids and their corresponding siRNAs or negative control (NC). (b) Effect of each endophilin isoform-specific siRNA on the expression of other endophilin isoforms in HEK293 cells. (c) Neurons cotransfected with GFP (green) and NC or Endo1 siRNA. Cultures were stained using an endophilin1 antibody (red). Scale bar, 20 *μ*m. (d) The percentages of endophilin-positive neurons with each treatment were quantified as the mean ± SEM of three independent experiments. ^*∗∗*^ *p* < 0.005.

The efficacy of the designed siRNAs against the endophilin isoforms was tested in HEK293 cells. At least one interference fragment selectively inhibited the expression of its corresponding endophilin (Figure 1(a)). The specificity of each endophilin isoform siRNA was also determined. As shown in Figure 1(b), isoform-specific siRNAs did not reduce the expression of other endophilin isoforms. We next confirmed the effectiveness of the siRNAs in cultured hippocampal neurons. As shown in Figure 1(c), compared with the NC, the expression of endogenous endophilin1 was significantly knocked down in the Endo1 siRNA-transfected neurons. Similar results for the siRNA effect were also observed with the other two endophilin isoforms (data not shown). Isoform-specific siRNA resulted in 70–80% knockdown of the corresponding endogenous endophilin (Figure 1(d)). These results indicate that the designed endophilin isoform-specific siRNAs are effective.

**Figure 2:**
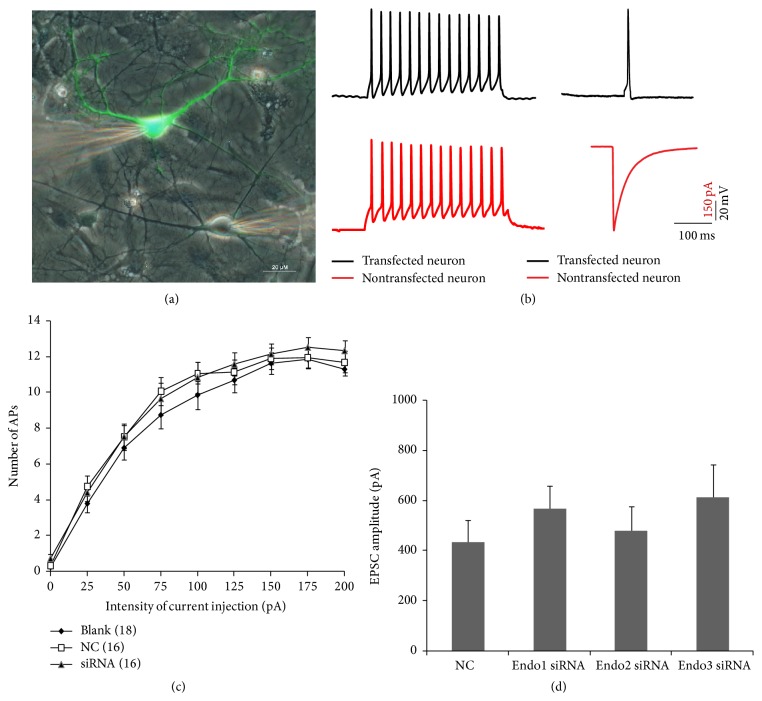
Basic electrophysiological characteristics and evoked excitatory postsynaptic currents (EPSCs) of transfected neurons. (a) Dual-cell patch-clamp recorded hippocampal neurons transfected with siRNA or a scrambled sequence (negative control, NC) (left, green) and nontransfected neurons (right, gray). (b) Representative traces from neurons. Transfected and nontransfected neurons exhibit many action potentials (APs) following 400 ms, 200 pA current injection (left panel). EPSC evoked in a nontransfected neuron by a single AP from a transfected neuron (right panel). (c) Input-output relationship of APs evoked by 400 ms current injection at various intensities in normal neurons (blank, *n* = 18), NC (*n* = 16), and siRNA-transfected neurons (*n* = 16, including five Endo1 siRNAs, five Endo2 siRNAs, and six Endo3 siRNAs). (d) EPSC amplitude evoked in a nontransfected neuron by a single AP in a neuron transfected with NC (*n* = 19), Endo1 siRNA (*n* = 13), Endo2 siRNA (*n* = 11), and Endo3 siRNA (*n* = 10).

To examine whether endophilin isoforms affected SV endocytosis, we performed dual-cell patch-clamp recordings in cultured hippocampal neurons. Neurons transfected with siRNA or NCs (Figure 2(a) left, green) were challenged with various stimulation patterns, and EPSCs were recorded from neighboring nontransfected cells (Figure 2(a) right, gray). We first assessed whether the intrinsic electrophysiological features of the transfected neurons were altered by transfection of siRNA or NC. The number of APs induced under the same stimulation parameters in neurons transfected with either siRNA (*n* = 16, including five Endo1 siRNAs, five Endo2 siRNAs, and six Endo3 siRNAs) or NC (*n* = 16) was consistent with that in nontransfected neurons (*n* = 18; *p* > 0.05; Figure 2(c)), indicating that introducing siRNA or NC into cultured neurons did not change the intrinsic electrophysiological features of neurons. When stimulating at a low frequency (0.1 Hz for 3 min), neurons transfected with either siRNA or NC released neurotransmitters in response to brief depolarizing pulses (Figure 2(b), right). The EPSC amplitude evoked from NC-transfected neurons was 435.492 ± 86.872 pA (*n* = 19). The EPSC amplitudes in endophilin isoform knockdown groups were 569.328 ± 89.497 pA for Endo1 siRNA (*n* = 13), 478.991 ± 97.81 pA for Endo2 siRNA (*n* = 11), and 615.125 ± 129.694 pA for Endo3 siRNA (*n* = 10), which were not significantly different from that in the NC group (*p* > 0.05, Figure 2(d)). These results suggest that knocking down endophilin isoforms has no apparent effect on SV exocytosis, consistent with a report that ablation of endophilin in* Drosophila* yields no significant evoked excitatory junctional potential changes [29].

### 3.2. Endophilin1 and Endophilin2, but Not Endophilin3, Are Involved in SV Endocytosis

**Figure 3:**
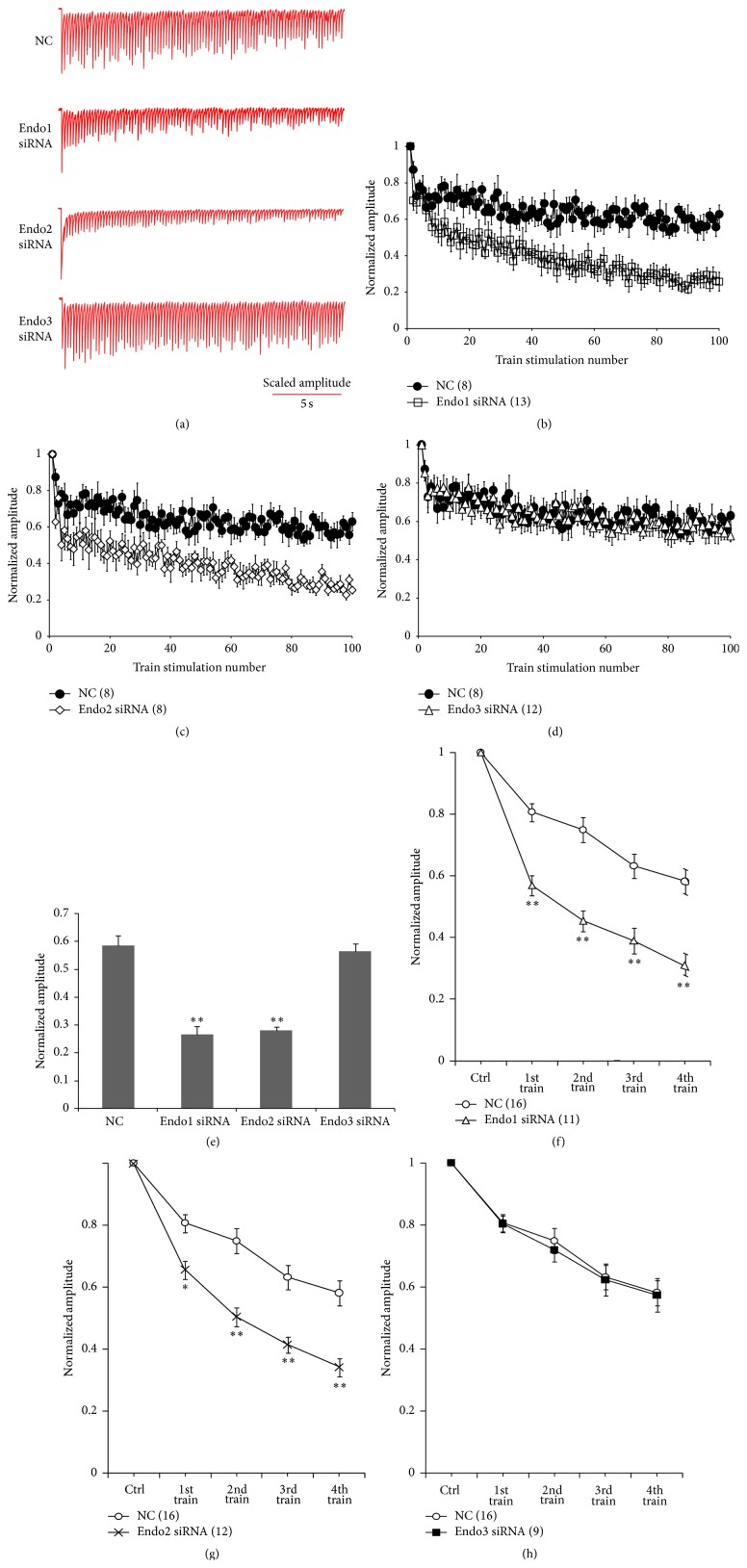
Quantitative analysis of endophilin isoform knockdown on sustained neurotransmitter release. (a) Representative traces of excitatory postsynaptic currents (EPSCs) in nontransfected neurons evoked by 100 pulses at 5 Hz in neurons transfected with control or endophilin siRNA. (b–d) Changes in short-term synaptic depression (STD) in nontransfected neurons evoked by stimulation of 100 pulses at 5 Hz in neurons transfected with endophilin siRNA ((b), Endo1 siRNA, *n* = 13; (c), Endo2 siRNA, *n* = 8; and (d), Endo3 siRNA, *n* = 12). (e) Average normalized amplitude of the last 20 responses in endophilin siRNA-transfected neurons compared with that in scrambled negative control- (NC-) transfected neurons; ^*∗∗*^ *p* < 0.005. (f–h) Normalized amplitude of EPSCs evoked by multiple train stimulations in neurons transfected with NC or endophilin siRNA. (f), Endo1 siRNA, *n* = 11; (g), Endo2 siRNA, *n* = 12; and (h), Endo3 siRNA, *n* = 9. ^*∗*^ *p* < 0.05, ^*∗∗*^ *p* < 0.005.

When stimulating at high frequency, a reduction in the synaptic response (called short-term synaptic depression, STD) was observed as a result of depleting the RRP of the SV (Figure 3(a), NC). At a stimulating intensity of 5 Hz (100 stimuli), synaptic depression was enhanced in neurons transfected with Endo1 siRNA compared with that in neurons transfected with NC (Figures 3(a) and 3(b)). Similar results were also observed for Endo2 siRNA-transfected neurons (Figures 3(a) and 3(c)). The marked STD in Endo1 siRNA- and Endo2 siRNA-transfected neurons suggested that SV endocytosis was impaired. These results were consistent with previous observations when SV endocytosis was impaired [27, 29]. However, the EPSC amplitude in Endo3 siRNA-transfected neurons was not decreased compared with that in NC-transfected neurons (Figures 3(a) and 3(d)). The mean normalized EPSC amplitudes for the last 20 responses in neurons transfected with Endo1 siRNA and Endo2 siRNA were 0.266 ± 0.029 (*n* = 13) and 0.281 ± 0.012 (*n* = 8), respectively, significantly lower than that in the NC group (0.586 ± 0.034, *n* = 8, ^*∗∗*^ *p* < 0.005). However, there was no significant difference in the mean normalized EPSC amplitude between the Endo3 siRNA (0.566 ± 0.027, *n* = 12) and NC groups (*p* > 0.05, Figure 3(e)).

When SV endocytosis is impaired, replenishment of the RRP is also hindered. Thus, to further confirm that these three endophilin isoforms played distinct roles in SV endocytosis, presynaptic neurons were challenged with consecutive high-frequency stimulation. Four trains of stimulation (5 Hz, 100 stimuli) were applied with a 3 min interval, during which time the presynaptic neuron was stimulated at 0.1 Hz. When multiple train stimulations were applied, a steady decrease was observed in the averaged EPSC amplitude evoked at 0.1 Hz during the interval between single train stimulations. Neurons transfected with Endo1 siRNA exhibited an accelerated decrease in the EPSC amplitude from the beginning of the 1st train to the 4th train compared with neurons transfected with NC (*n* = 11, ^*∗∗*^ *p* < 0.005; Figure 3(f)). A similar result was also found in the Endo2 siRNA group (*n* = 12, ^*∗*^ *p* < 0.05 after the 1st train, ^*∗∗*^ *p* < 0.005 after the 2nd, 3rd, and 4th trains; Figure 3(g)). However, no significant difference was detected in the EPSC amplitude between the Endo3 siRNA group and the NC group (*n* = 9, *p* > 0.05; Figure 3(h)). These electrophysiological results suggest that replenishment of the RRP is hindered in Endo1 siRNA- and Endo2 siRNA-transfected neurons but not in Endo3 siRNA-transfected neurons.

### 3.3. Endophilin1 and Endophilin2 Function Together, Not Independently, in SV Endocytosis

**Figure 4:**
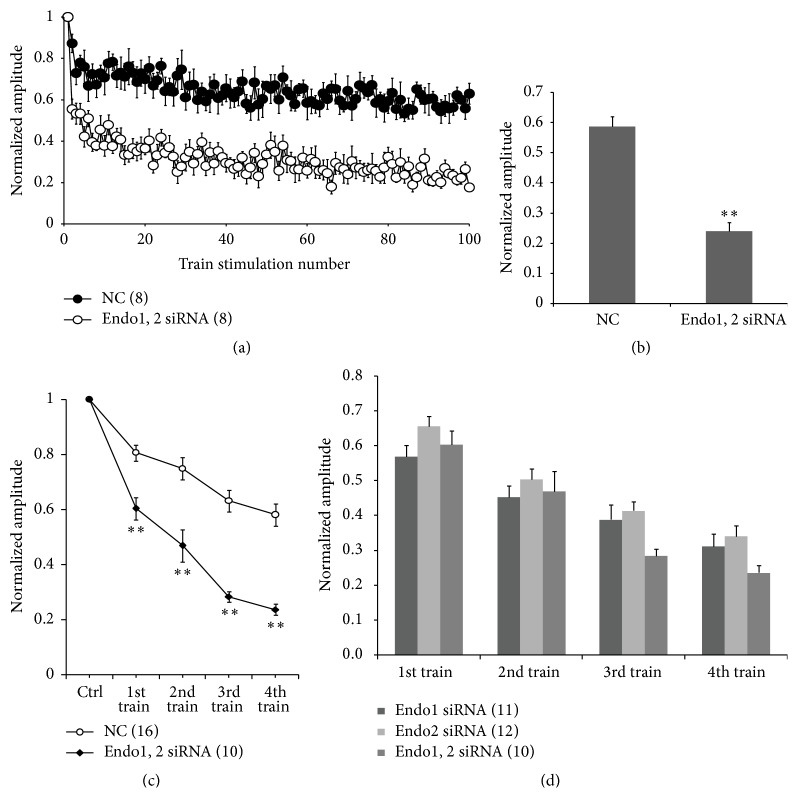
Quantitative analysis of sustained neurotransmitter release in endophilin1, endophilin2 (Endo1, 2) double-knockdown neurons. (a) Changes in short-term synaptic depression (STD) in nontransfected neurons evoked by 100 pulses at 5 Hz in neurons cotransfected with Endo1 and Endo2 siRNAs (*n* = 8). (b) Average normalized amplitude of the last 20 responses in Endo1, 2 siRNA-transfected and scrambled negative control- (NC-) transfected neurons; *n* = 8, ^*∗∗*^ *p* < 0.005. (c) Normalized amplitude of excitatory postsynaptic currents (EPSCs) evoked by multiple train stimulation in neurons transfected with control or Endo1, 2 siRNA; ^*∗∗*^ *p* < 0.005. (d) Normalized amplitudes compared among Endo1 siRNA (*n* = 11), Endo2 siRNA (*n* = 12), and Endo1, 2 siRNA (*n* = 10) after the 1st, 2nd, 3rd, and 4th train stimulation; *p* > 0.05.

To investigate the relationship between endophilin1 and endophilin2 in SV endocytosis, neurons cotransfected with Endo1 siRNA and Endo2 siRNA (Endo1, 2 siRNA) were stimulated using single high-frequency stimulation and consecutive high-frequency stimulation. The response of the Endo1, 2 siRNA knockdown neurons during a single high-frequency stimulation revealed that the EPSC amplitude rapidly decreased to reach a significantly lower plateau (approximately 30% of the control) compared with that in the NC group (*n* = 8, Figure 4(a)). The mean normalized EPSC amplitude for the last 20 responses in the Endo1, 2 siRNA group was significantly lower than that in the control group (0.240 ± 0.023 versus 0.586 ± 0.034, *n* = 8, ^*∗∗*^ *p* < 0.005; Figure 4(b)). However, no significant difference was detected for this measure among the Endo1 siRNA, Endo2 siRNA, and Endo1, 2 siRNA groups (Figures 3(e) and 4(b)). When given multiple train stimulations, neurons transfected with Endo1, 2 siRNA exhibited an accelerated decrease in the EPSC amplitude from the beginning of the 1st train to the 4th train compared with neurons in the NC group (*n* = 10, ^*∗∗*^ *p* < 0.005; Figure 4(c)). The normalized EPSC amplitudes after the 1st and 2nd trains in the Endo1, 2 siRNA cotransfected group were consistent with those in both Endo1 siRNA and Endo2 siRNA groups. Although the Endo1, 2 siRNA group exhibited lower EPSC amplitudes than either Endo1 siRNA or Endo2 siRNA groups after the 3rd and 4th trains, there was no significant difference among them (*p* > 0.05, Figure 4(d)). These results suggest that endophilin1 and endophilin2 influence SV endocytosis, functioning together, but not independently, to mediate SV endocytosis.

## 4. Discussion

In our previous study, we found that all endophilin isoforms formed endophilin-Ca^2+^ channel complexes in neurons. Endophilin1 and endophilin2 demonstrated clear Ca^2+^-dependent interactions with the Ca^2+^ channel, whereas endophilin3 did not [26]. However, it remained unknown whether this characteristic would affect SV endocytosis. To address this issue in the present study, we generated endophilin isoform-specific knockdowns in cultured hippocampal neurons. Each endophilin siRNA successfully reduced the expression of its corresponding isoform approximately 70–80% (Figure 1). Neither transfected siRNA nor NC affected the tested intrinsic electrophysiological properties of the neurons. As shown in Figure 2(d), the EPSC amplitude evoked at low frequency in each siRNA group exhibited no significant difference from that in neurons transfected with NC, suggesting that none of the endophilin isoform knockdowns affected SV exocytosis, consistent with results from a study that ablated endophilin in* Drosophila* [29].

By microinjecting anti-endophilin antibodies into the giant axon of the lamprey, scientists first obtained evidence that endophilin is involved in SV endocytosis [10]. Fly and worm mutants lacking endophilin also demonstrated that endophilin is an important part of the machinery driving SV recycling [29, 30]. Using cultured cortical neurons derived from endophilin triple knockout (TKO) mice, Milosevic et al. showed that endophilin was implicated in SV endocytosis at mammalian central synapses, particularly in the process of clathrin uncoating [9]. In our study, through selectively knocking down endophilin isoforms in cultured hippocampal neurons, we found that neurons with endophilin1 or endophilin2 knockdowns exhibited synaptic depression, similar to the results in flies and TKO mice [9, 29], whereas neurons with the endophilin3 knockdown were not different from control neurons in this regard. This result demonstrates that SV endocytosis is sustained by endophilin1 or endophilin2 isoforms, but not by endophilin3. Although all three endophilins contain BAR and SH3 domains, which are considered the molecular basis of the endophilin involvement in SV endocytosis [13, 23], they do not exhibit the same functions in SV endocytosis. This may be related to differences in their variable regions between the BAR and SH3 domains. Previous analysis on the secondary structure of the proteins indicated that the variable regions in endophilin1 and endophilin2 carry short *α*-helices, whereas a *β*-turn was detected in the N-terminal region of endophilin3 [20]. Our previous study showed that the variable regions of endophilin1 and endophilin2 also harbor a calcium binding site (E264), but no calcium binding site was found in endophilin3 [26]. The calcium binding site enables endophilin to interact with other proteins at resting Ca^2+^ levels and dissociate at high Ca^2+^ levels [25, 27]. In the present study, compared with endophilin3, the amplitude of the EPSC evoked from endophilin1 or endophilin2 isoform-specific knockdown neurons rapidly decreased after stimulation (Figure 3). In this situation, neurons are depolarized and the Ca^2+^ concentration is considered to be at a high level (over 1 *μ*M). These data suggest that although the BAR and SH3 domains are necessary for the role of endophilin in SV endocytosis, the contribution of the calcium binding sites in endophilin1 and endophilin2 may be more important for endophilin function.

Reducing the endogenous levels of either endophilin1 or endophilin2 through RNA inference impedes SV endocytosis (Figure 3). However, the response of cotransfected Endo1, 2 siRNA neurons to single high-frequency or multiple high-frequency stimulation displayed no obvious difference compared with those of neurons transfected with Endo1 or Endo2 siRNA alone. This suggests that the two endophilins do not have overlapping roles in SV endocytosis. Studies have shown that endophilin1 and endophilin2 are found predominantly as stable dimers through a coiled-coil domain in their conserved NH2-terminal moiety [31]. This suggests that endophilin1 and endophilin2 may function together, not independently, to mediate SV endocytosis. Dimerization may allow endophilins to link a number of different cellular targets to the endocytic machinery.

In conclusion, our study suggests that, in cultured mammalian primary neurons, endocytosis of presynaptic vesicles is sustained by endophilin1 or endophilin2 isoforms, but not by endophilin3, suggesting that isoform specificity confers particular properties on specific endocytic pathways. The mechanism for this isoform specificity remains to be determined in future studies.
